# Influence of exposure parameters and iterative reconstruction on automatic airway segmentation and analysis on MDCT—An *ex vivo* phantom study

**DOI:** 10.1371/journal.pone.0182268

**Published:** 2017-08-02

**Authors:** Patricia Leutz-Schmidt, Oliver Weinheimer, Bertram J. Jobst, Julien Dinkel, Jürgen Biederer, Hans-Ulrich Kauczor, Michael U. Puderbach, Mark O. Wielpütz

**Affiliations:** 1 Department of Diagnostic and Interventional Radiology, University Hospital of Heidelberg, Heidelberg, Germany; 2 Translational Lung Research Center (TLRC) Heidelberg, Member of the German Center for Lung Research (DZL), Heidelberg, Germany; 3 Department of Diagnostic and Interventional Radiology with Nuclear Medicine, Thoraxklinik at University of Heidelberg, Heidelberg, Germany; 4 Radiologie Darmstadt, Gross-Gerau County Hospital, Gross-Gerau, Germany; 5 Department of Radiology, German Cancer Research Center (dkfz), Heidelberg, Germany; Institute of Lung Biology and Disease (iLBD), Helmholtz Zentrum München, GERMANY

## Abstract

**Objectives:**

To evaluate the influence of exposure parameters and raw-data-based iterative reconstruction (IR) on computer-aided segmentation and quantitative analysis of the tracheobronchial tree on multidetector computed tomography (MDCT).

**Material and methods:**

10 porcine heart-lung-explants were mounted inside a dedicated chest phantom. MDCT was performed at 120kV and 80kV with 120, 60, 30 and 12 mAs each. All scans were reconstructed with filtered back projection (FBP) or IR, resulting in a total of 160 datasets. The maximum number of detected airway segments, most peripheral airway generation detected, generation-specific airway wall thickness (WT), total diameter (TD) and normalized wall thickness (pi10) were compared.

**Results:**

The number of detected airway segments decreased slightly with dose (324.8±118 at 120kV/120mAs vs. 288.9±130 at 80kV/30mAs with FBP, p<0.05) and was not changed by IR. The 20^th^ generation was constantly detected as most peripheral. WT did not change significantly with exposure parameters and reconstruction algorithm across all generations: range 1^st^ generation 2.4–2.7mm, 5^th^ 1.0–1.1mm, and 10^th^ 0.7mm with FBP; 1^st^ 2.3–2.4mm, 5^th^ 1.0–1.1mm, and 10^th^ 0.7–0.8mm with IR. pi10 was not affected as well (range 0.32–0.34mm).

**Conclusions:**

Exposure parameters and IR had no relevant influence on measured airway parameters even for WT <1mm. Thus, no systematic errors would be expected using automatic airway analysis with low-dose MDCT and IR.

## Introduction

Chronic smoking-related respiratory diseases, mainly chronic obstructive pulmonary disease (COPD), are of high prevalence with a high morbidity and mortality worldwide. About 5% of Europe’s population suffer from COPD [1] and it is predicted to become one of the most common reasons for death in adults in 2020 [2]. To fully define the extent of regional lung damage, accurate structural imaging is important and may identify distinct phenotypes such as “airways dominant” and “emphysema dominant” [3, 4]. For this, multidetector computed tomography (MDCT) is the method of choice, which also offers options for further quantitative post-processing of imaging biomarkers [5–7]. Airway wall thickness and low attenuation areas of lung tissue are key biomarkers and correlate well with a reduction of lung function in COPD [5, 6, 8–10]. To reduce ionizing radiation, low-dose MDCT may be performed for imaging COPD, but it entails an increase in noise with potential influence on quantitative post-processing. Iterative reconstruction (IR) techniques, as they are available from different CT vendors, result in noise reduction compared to filtered back-projection (FBP) and potentially contribute to further reduction of radiation dose [11, 12], while maintaining diagnostic image quality and contrast-to-noise ratio (CNR) [13, 14]. However, so far there is little data available on the influence of IR onto quantitative CT of the airways and potential systematic errors are discussed controversely [15, 16]. Thus, the present study was conducted to determine the potential effects of exposure parameters and IR algorithms on quantitative airway analysis with low dose MDCT. For this purpose, we used an *ex vivo* system with porcine lung explants inside a chest phantom for repetitive MDCT scanning with variable parameters and standardized airway analysis with well-evaluated post-processing software [17–19].

## Materials and methods

### The ex vivo lung phantom

**Fig 1:**
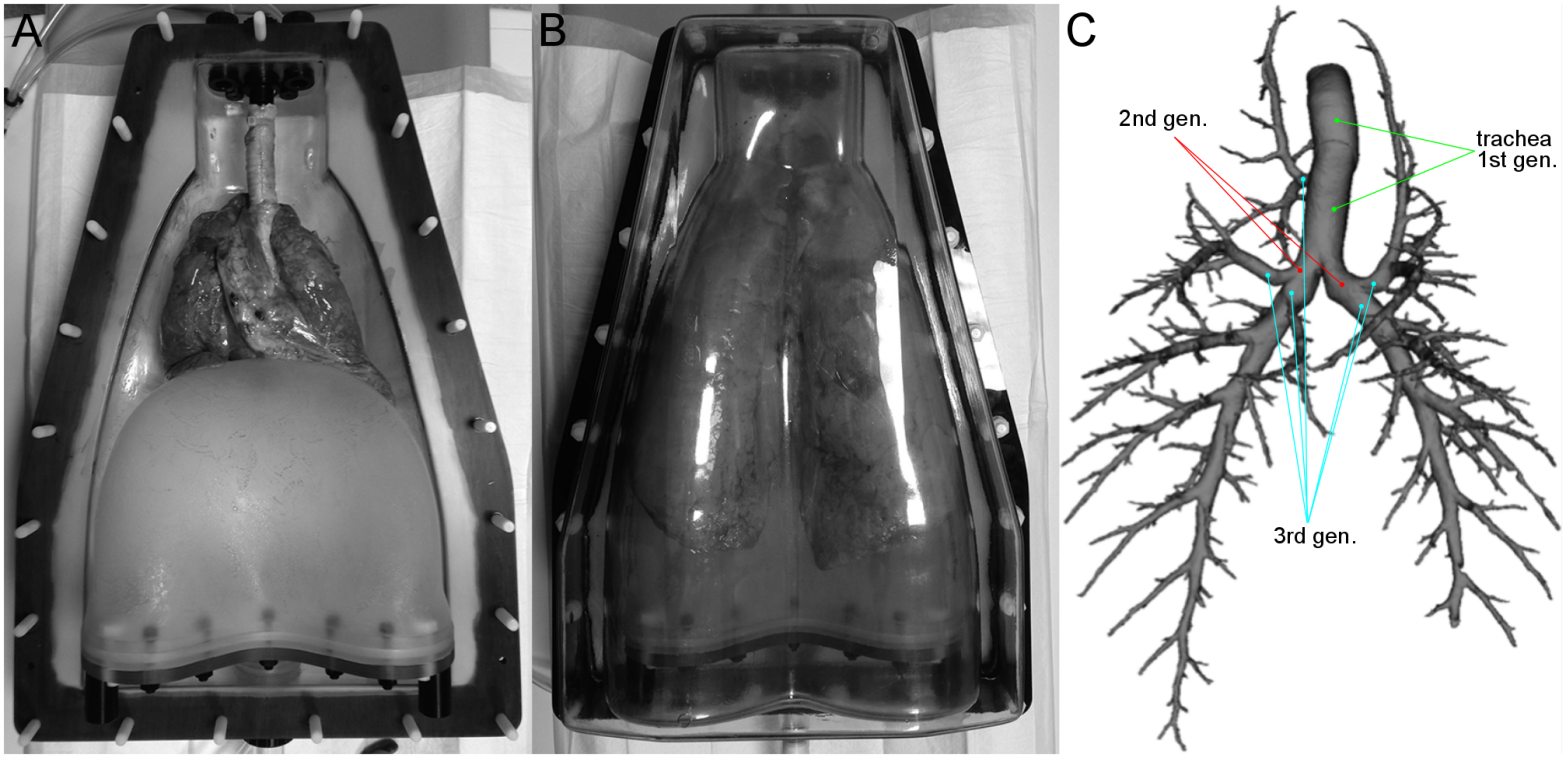
Porcine heart-lung-explant (A) was inflated with room air in an artificial shell (B). Continuous negative pressure was held up to mimic artificial pleural space. After MDCT, Data were sent directly to the post-processing server. The result of airway segmentation is shown as a three-dimensional volume reconstruction. The trachea is assigned to generation 1, right and left main bronchi are assigned to generation 2, tracheal bronchus starts at generation 3. That means we count 1 airway at generation 1, 2 at generation 2, 5 at generation 3, 10 at generation 4, and further 5*2^(i-3) airways for generation i, i>2 (C).

We used a commercially available *ex vivo* system for imaging studies with porcine heart-lung-explants (Artichest, PROdesign GmbH, Heiligkreuzsteinach, Germany) [20–22] (Fig 1A and 1B). Ten heart-lung-explants from mature standard domestic pigs were obtained from a local slaughterhouse in Mannheim, Germany (Fleischversorgungszentrum Mannheim, Schlachthofstr. 21, 68165 Mannheim). Careful attention was paid to harvest lungs with an intact surface to avoid air leakage. No animal was sacrificed for the particular purpose of this study and thus, no ethics committee approval was required.

### Multidetector computed tomography

MDCT scans were acquired with a Somatom Definition Flash computer tomograph (Siemens Medical Solutions AG, Forchheim, Germany). Calibration for water and for air was done regularly. Each of the 10 lungs was scanned 8 consecutive times. The collimation was kept constant at 128 × 0.6 mm and tube potentials of 120 and 80 kV were combined with different tube currents of 120, 60, 30, and 12 mAs. As published previously, these parameters equal to CT dose indices (CTDI) from a maximum of 8.07 mGy to a minimum of 0.25 mGy [21, 22].

For each scan, data were reconstructed with a medium-soft FBP (B40f) kernel and corresponding IR (I40f) kernel at identical slice thickness (0.75 mm), slice increment (0.6 mm), matrix (512^2^) and field-of-view (300 × 300 mm), essentially as previously described [21, 22]. IR was performed with raw-data based algorithm (Safire, Siemens Medical Solutions AG, Forchheim, Germany). “Strength” 3 (strength 1–5 are available) was selected as proposed by previous studies [21, 23]. Thus, 16 datasets for each lung with 160 datasets in total were generated. We have shown previously with identical conditions in this phantom setup that IR “strength” 3 reduced noise by approx. 25% on average at each combination of exposure parameters [21, 22].

### Quantitative post-processing

YACTA (version 2.5.4.3) was used in a fully automatic server mode as previously described [17, 19, 24–27]. YACTA is a non-commercial software for scientific purpose (please refer to Oliver.Weinheimer@med.uni-heidelberg.de). The software’s accuracy for airway dimension measurements has extensively been validated in previous studies by using 1.) tube phantoms [17], 2.) histology in pigs [26], 3.) computational phantoms, and 4.) microCT in pigs [28]. Further, reproducibility of the measurements has been confirmed in pigs with repeated scans [17]. The software has special rules to correctly determine the generations of the human airway tree up to the lobar level (3rd generation), for higher generations a dichotomic branching pattern is taken as basis, the generation number is increased by 1 after each branching (bifurcation) detected. As a concession to the pig phantom the automatic landmark detection algorithms could not be used and it was necessary to manually mark the trachea and the tracheal bifurcation once per lung, before starting the automatic evaluation. The complete trachea is assigned to generation number one, right and left main bronchi are assigned to generation two, tracheal bronchus starts at generation three. For all other airways the generation number is increased by one after each branching (Fig 1C).

YACTA applied a Gaussian filter on the image data. Then airway tree segmentation is applied followed by skeletonization and graph generation algorithms. The branchings of the bronchi correspond to the nodes of the graph. The direction vector of an airway can be determined by the graph representation of the airway tree. Hence, it is possible to calculate an orthogonal plane for every airway, more precisely for every skeleton point between two nodes. A modified version of the previously validated integral based method (IBM) [17] was used for airway geometry measurement on virtual rays directed from the center of the airway in the calculated orthogonal plane. The airway wall can then be determined on the density profiles corresponding to the rays. The number of airways per generation as well as the most peripheral airway generation detected were recorded for each reconstruction. The following quantitative parameters were calculated: airway wall thickness (WT) and total diameter (TD) for each airway generation, as well as the normalized wall thickness index (pi10) as previously described for COPD [29]. The average distance from outer to outer border of the airway wall determines the TD of an airway segment. WT is defined as the average distance between inner and outer airway wall border, and corresponding WT% is calculated as WT/TD x 100 [29].

### Statistical analysis

Data were compiled with Excel (Microsoft Corp., Redmond, USA) and analyzed with SigmaPlot (Systat Software GmbH, Erkrath, Germany) software. Repeated measures analysis of variance (ANOVA on ranks) was used for comparison within the groups in FBP and IR. Wilcoxon signed rank test was used to compare FBP vs. IR. Significance at individual p-values < 0.05 are reported only when significance is maintained after adjustment for multiple comparisons by the Bonferroni-Holm method [30]. The difference between measured airway parameters was assessed with the method of Bland and Altman also [31].

## Results

All 10 porcine lungs could be analyzed by YACTA. There was a tendency to less airway segments detected at a higher standard deviation with decreasing tube potential and current. For example, the mean number of detected airway segments was 324.8±118 for 120 kV 120 mAs and 288.9±130 for 80 kV 30 mAs with FBP (Table 1). Especially also the number of possible measurements for airway segments (detected airway segments x length) was higher at 120 kV 120 mAs 4268.3 ± 914.7 compared to 80 kV 12 mAs with a mean of 3781.9 ± 1093.3 respectively (data not shown).

**Table 1:** Averaged airway parameters grouped according to exposure and reconstruction parameters.

	kV	mAs	CTDI (mGy)	WT%	Measurements	WT% TD 8 → 3 mm	Pi10 (mm)	Most distal generation
**FBP**	**120**	**120**	8.07	44.2±7.4	324.8±118#	45.3±7.6	0.33±0.04	19.9±3.5
		**60**	4.06	43.5±7.0	307.5±117#	44.6±7.2	0.32±0.04	19.5±3.27
		**30**	2.03	43.2±7.0	307±112	44.4±7.1	0.32±0.04	19.7±3.3
		**12**	0.86	43.6±6.9	309.2±133	44.8±7.2	0.33±0.04	19.7±4.3
	**80**	**120**	2.35	43.6±7.1	301.3±115	44.7±7.3	0.32±0.04	19.2±3.52
		**60**	1.17	43.8±7.2	312.1±124	44.9±7.4	0.33±0.04	19.4±3.94
		**30**	0.59	43.2±7.0	288.9±130	44.5±7.2	0.33±0.04	20.1±5.52
		**12**	0.25	43.9±6.9	311.5 ±165	45.3±7.2#	0.34±0.04#	20.0±4.49
**IR**	**120**	**120**	8.07	43.9±7.4*	326.5±122#	45.1±7.6*	0.32±0.04*	19.9±3.4
		**60**	4.06	43.3±6.9	324.6±145#	43.4±7.4*	0.32±0.03	18.3±3.1
		**30**	2.03	44.4±5.9	309±116	45.5±6.0*	0.33±0.01	19.6±3.4
		**12**	0.86	43.5±7.0	318.5±130	44.7±7.3	0.32±0.04	19.7±3.7
	**80**	**120**	2.35	43.4±7.1	302.7±118	44.5±7.2*	0.32±0.04	19.3±3.1
		**60**	1.17	43.6±7.2	320.6±133	44.7±7.4*	0.32±0.04	19.4±3.7
		**30**	0.59	43.1±7.0	298.4±137	44.3±7.2	0.33±0.04	20.1±5.7
		**12**	0.25	43.7±7.0	375.1±200	45.1±7.2*	0.34±0.04	21.8±6.6

FBP = filtered back-projection, IR = iterative reconstruction.

Data presented as mean ± standard deviation.

* p<0.05 vs. corresponding exposure settings with FBP.

^#^ p<0.05 vs. reference 120 kv 120 mAs with FBP or IR group (p-values for tests vs. other exposure parameters than reference not shown for sake of clarity).

In a subgroup analysis for airways with TD 8 → 3 mm, which compare to lobar, segmental and proximal subsegmental airways in humans, we made similar observations. Thus, the reduced number of detected segments is not solely due to failed segmentation of most peripheral airways (Table 1). Supporting this notion, the most distal airway generation detected on average remained constantly at 20^th^ generation, except for 80 kV 12 mAs with IR, for which it was even 22^nd^ generation (p<0.05) (Table 1).

### Influence of exposure parameters on airway dimensions

Because WT% averaged across all airways detected, was stable amongst all exposure parameters, despite the slightly reduced number of detected airway segments with decreasing radiation dose, we grouped airways with a TD from 8 → 3 mm for a combined analysis. Even in this clinically important fraction of airways we could show that WT% is unchanged by varying exposure parameters (Table 1). Another gross indicator of wall thickness, pi10, also was not affected by a reduction of exposure parameters with one exception: at 80 kV 12 mAs it was significantly higher by 0.01 mm on average compared to 120 kV 120–30 mAs FBP (p<0.05).

**Fig 2:**
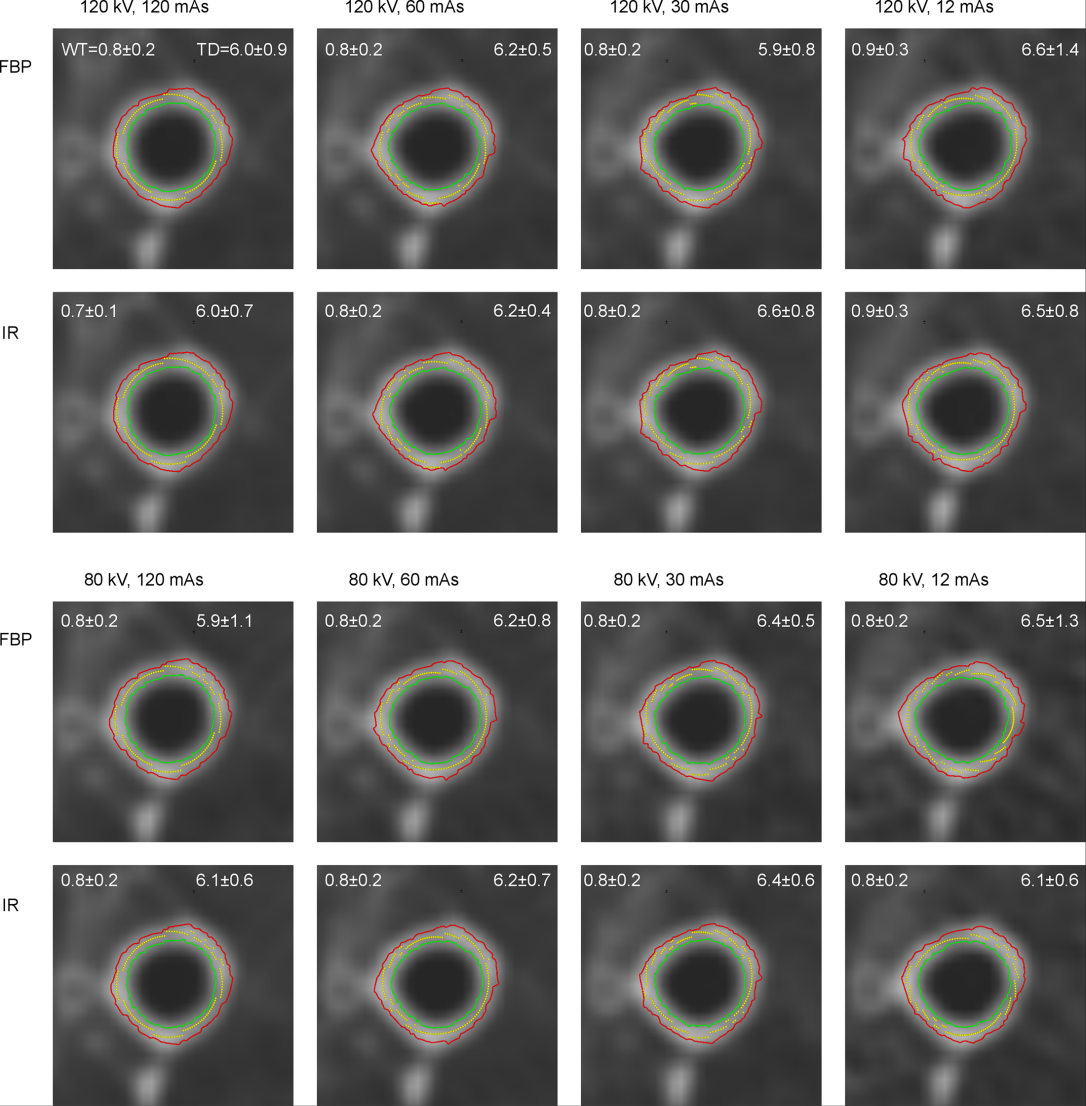
Representative images showing the identical 7^th^ generation subsegmental airway in all series acquired, and reconstructed with either filtered back-projection (FBP top line) or iterative reconstruction (IR, bottom line). Corresponding wall thickness (top left corner) and total diameter (top right corner) is given at the top of each image.

In a generation-based approach, WT was not significantly different within the FBP group comparing all combinations of exposure parameters, except for few outliers. For example, WT for 1^st^ generation was 2.4 ± 0.3 mm with 120 kV 120 mAs which compares to 2.4 ± 0.4 mm for lowest tube potential and current 80 kV 12 mAs FBP. Even for more distal airways below 1 mm of wall thickness it remained unaffected (Fig 2) and also for example for the 15^th^ generation WT was 0.7 ± 0.2 mm at 120 kV 120 mAs and 0.8 ± 0.3 mm at 80 kV 12 mAs FBP (Tables 2 and 3). Similarly, there was also no significant difference for WT% from 1^st^—20^th^ airway generation and all exposure settings (data not shown).

**Table 2:** Wall thickness for 1^st^ to 10^th^ airway generation (mm).

	[kV]		1st	2nd	3rd	4th	5th	6th	7th	8th	9th	10th
**FBP**	**120**	**120**	2.4±0.3	2.2±0.4	2.0±0.3	1.5±0.4	1.0±0.3	0.9±0.8	0.8±0.2	0.8±0.2	0.7±0.1	0.7±0.2
		**60**	2.4±0.4	2.2±0.4	2.0±0.4	1.4±0.4	1.1±0.3	0.9±0.2	0.8±0.2	0.7±0.1	0.7±0.1	0.7±0.1
		**30**	2.3±0.6	2.0±0.6	1.9±0.4	1.3±0.3	1.0±0.3	0.8±0.2	0.8±0.2	0.8±0.2	0.7±0.1	0.7±0.2
		**12**	2.3±0.3	2.1±0.4	2.0±0.4	1.4±0.4	1.0±0.3	0.9±0.3	0.9±0.3	0.8±0.2	0.8±0.3	0.7±0.2
	**80**	**120**	2.4±0.4	1.9±0.3	1.9±0.3	1.3±0.2	1.1±0.3	0.9±0.2	0.8±0.2	0.8±0.2	0.7±0.1	0.7±0.2
		**60**	2.3±0.3	2.2±0.4	2.0±0.3	1.4±0.4	1.1±0.3	0.9±0.3	0.8±0.2	0.8±0.2	0.7±0.2	0.7±0.2
		**30**	2.4±0.3	2.2±0.4	2.0±0.4	1.4±0.3	1.0±0.3	0.9±0.3	0.8±0.2	0.7±0.2	0.7±0.2	0.8±0.2
		**12**	2.4±0.4	2.3±0.3	1.8±0.4	1.4±0.3	1.1±0.3	0.3±0.3	0.8±0.2	0.8±0.2	0.8±0.2	0.7±0.2
**IR**	**120**	**120**	2.3±0.4	2.2±0.4	1.9±0.3	1.3±0.2*	1.1±0.3	0.8±0.2	0.7±0.1*	0.8±0.2	0.7±0.1	0.7±0.0
		**60**	2.4±0.4	2.2±0.4	1.9±0.4	1.3±0.2	1.1±0.3	0.8±0.2	0.8±0.2	0.7±0.1	0.7±0.1	0.7±0.2
		**30**	2.4±0.3	2.3±0.3	2.0±0.3	1.4±0.4	1.2±0.5	0.9±0.2	0.8±0.2	0.8±0.2	0.7±01	0.7±0.1
		**12**	2.4±0.4	2.2±0.4	1.9±0.3	1.4±0.3	1.1±0.3	0.9±0.2	0.8±0.2	0.8±0.2	0.7±0.2	0.7±0.1
	**80**	**120**	2.4±0.4	2.1±0.4	2.0±0.3	1.3±0.2	1.1±0.3	0.8±0.2	0.8±0.2	0.7±0.1	0.7±0.1	0.7±0.2
		**60**	2.3±0.3	2.2±0.4	1.9±0.3	1.4±0.4	1.1±0.3	0.9±0.3	0.8±0.2	0.8±0.2	0.7±0.2	0.7±0.2
		**30**	2.3±0.4	2.2±0.3	1.9±0.3	1.4±0.3	1.0±0.3	0.9±0.2	0.8±0.2	0.7±0.1	0.7±0.1	0.7±0.2
		**12**	2.4±0.4	2.3±0.5	1.9±0.5	1.5±0.5	1.1±0.4	1.0±0.4	0.8±0.2	0.8±0.2	0.8±0.2	0.8±0.2

FBP = filtered back-projection, IR = iterative reconstruction.

Data presented as mean ± standard deviation.

* p<0.05 vs. corresponding exposure settings with FBP.

^#^ p<0.05 vs. reference 120 kv 120 mAs with FBP or IR group (p-values for tests vs. other exposure parameters than reference not shown for sake of clarity).

**Table 3:** Wall thickness for 11^th^ to 20^th^ airway generation (mm).

	[kV]	mAs	11th	12h	13th	14th	15th	16th	17th	18th	19th	20th
**FBP**	**120**	**120**	0.7±0.1	0.7±0.2	0.6±0.2	0.7±0.1	0.7±0.2	0.7±0.3	0.6±0.2	0.7±0.2	0.7±0.2	0.6±0.2
		**60**	0.7±0.1	0.7±0.1	0.7±0.2	0.7±0.2	0.8±0.3	0.6±0.2	0.6±0.2	0.6±0.2	0.7±0.2	0.7±0.2
		**30**	0.7±0.1	0.6±0.1	0.7±0.2	0.7±0.2	0.8±0.3	0.6±0.2	0.6±0.2	0.7±0.2	0.7±0.2	0.7±0.2
		**12**	0.8±0.2	0.8±0.2	0.7±0.2	0.7±0.2	0.8±0.3	0.8±0.4	0.6±0.2	0.6±0.2	0.6±0.2	0.5±0.1
	**80**	**120**	0.6±0.1	0.7±0.2#	0.7±0.2	0.7±0.2	0.7±0.2	0.7±0.3	0.6±0.2	0.6±0.2	0.7±0.3	0.7±0.2
		**60**	0.7±0.1	0.7±0.2	0.6±0.1	0.7±0.2	0.7±0.2	0.7±0.2	0.7±0.3	0.6±0.2	0.6±0.2	0.6±0.2
		**30**	0.7±0.2	0.7±0.2	0.7±0.2	0.7±0.2	0.7±0.2	0.7±0.3	0.8±0.4	0.6±0.3	0.7±0.2	0.8±0.3
		**12**	0.8±0.2	0.8±0.2	0.7±0.2	0.7±0.2	0.8±0.3	0.8±0.4#	0.6±0.2	0.6±0.2	0.6±0.2	0.5±0.1
**IR**	**120**	**120**	0.7±0.2	0.7±0.2	0.6±0.1	0.6±0.2	0.6±0.2	0.6±0.2	0.7±0.3	0.6±0.1	0.7±0.3	0.7±0.2
		**60**	0.6±0.1	0.7±0.2	0.7±0.2	0.7±0.2	0.7±0.2	0.6±0.2	0.6±0.2	0.6±0.2	0.7±0.2	0.7±0.1
		**30**	0.7±0.2	0.7±0.2	0.6±0.2	0.7±0.2	0.8±0.2	0.8±0.3	0.6±0.2	0.7±0.2	0.7±0.2	0.7±0.2
		**12**	0.7±0.2	0.7±0.2	0.7±0.1	0.6±0.2	0.7±0.3	0.7±0.3	0.7±0.2	0.7±0.3	0.6±0.2	0.7±0.3
	**80**	**120**	0.6±0.1	0.6±0.2	0.7±0.2	0.7±0.2	0.6±0.2	0.7±0.2	0.8±0.3	0.6±0.2	0.7±0.3	0.6±0.2
		**60**	0.7±0.2	0.7±0.2	0.7±0.1	0.7±0.2	0.7±0.2	0.7±0.3	0.6±0.2	0.6±0.2	0.7±0.2	0.7±0.3
		**30**	0.7±0.2	0.7±0.2	0.7±0.2	0.7±0.3	0.7±0.2	0.7±0.3	0.7±0.4	0.7±0.2	2.6±4.4	0.7±0.2
		**12**	0.7±0.2	0.8±0.2	0.7±0.2	0.7±0.2	0.7±0.2	0.7±0.2	0.7±0.2	0.7±0.2	0.7±0.3	0.6±0.1

FBP = filtered back-projection, IR = iterative reconstruction.

Data presented as mean ± standard deviation.

* p<0.05 vs. corresponding exposure settings with FBP.

^#^ p<0.05 vs. reference 120 kv 120 mAs with FBP or IR group (p-values for tests vs. other exposure parameters than reference not shown for sake of clarity).

Similar results were obtained for TD. For example, TD remained constant at 27.8 ± 1.7 mm for 1^st^ airway generation at 120 kV 120 mAs and 26.7 ± 2.4 for 80 kV 120 mAs FBP, which equals to a difference of 3.96% for the mean TD values between these two settings. For the 15^th^ generation it was 5.9 ± 1.0 mm at 120 kV 120 mAs and 6.5 ± 1.0 mm at 80 kV 12 mAs (Tables 4 and 5), which means a difference of 9.23% for the mean TD values between these two settings.

**Table 4:** Total diameter for 1^st^ to 10^th^ airway generation (mm).

	[kV]	mAs	1st	2nd	3rd	4th	5th	6th	7th	8th	9th	10th
**FBP**	**120**	**120**	27.8±1.7	22.7±4.5	17.2±2.0	11.5±1.9	8.3±1.1	6.8±1.2	6.0±0.9	6.0±0.5	5.8±0.7	5.7±0.6
		**60**	26.8±3.5	22.8±4.1	17.6±3.1	11.3±2.7	8.9±1.5	6.4±1.2	6.2±0.5	6.1±0.6	5.8±0.7	5.7±0.5
		**30**	26.7±2.8	22.3±3.5	16.3±2.6	10.4±1.7	8.0±1.5	6.6±0.4	5.9±0.8	6.2±0.8	5.8±0.6	6.0±0.5
		**12**	26.9±1.4	23.6 ±2.9	17.8±3.6	9.7±4.0	8.9±2.3	7.3±1.7	6.6±1.4	6.4±1.6	6.4±1.7	6.1±1.1
	**80**	**120**	27.1±3.1	22.7±3.6	17.1±1.8	10.8±1.7	8.5±1.3	6.8±0.6	5.9±1.1	6.3±0.8	5.9±0.6	6.2±1.1
		**60**	26.8±1.5	23.4±2.7	17.7±2.9	11.1±2.4	8.5±1.6	6.9±0.7	6.2±0.8	6.2±0.6	5.6±0.5	6.3±0.8
		**30**	27.0±2.9	22.8±3.6	17.7±2.2	11.1±1.1	8.4±1.3	6.8±0.7	6.4±0.5	6.1±0.4	6,9±1.0	0.8±0.2
		**12**	26.7±2.4	23.6±1.6	15.5±3.2	11.4±1.9	9.0±1.8	7.3±1.2	6.5±1.3	6.6±1.2	6.1±0.5	6.0±0.6
**IR**	**120**	**120**	25.8±3.4	22.0±3.0	15.2±1.1*	9.8±1.7*	8.5±1.4	6.3±0.5	6.0±0.7	5.8±0.7	5.8±0.5	5.5±0.3
		**60**	27.0±3.2	22.3±3.6	16.4±1.4	10.6±0.2	8.2±1.6	6.6±0.4	6.2±0.4	6.1±0.6	5.7±0.6	6.0±7.7
		**30**	27.5±2.0	24.5±2.8	18.3±4.1	11.6±4.7	8.5±3.6	7.0±1.2	6.6±0.8	6.2±0.6	5.9±0.6	6.0±0.6
		**12**	26.9±3.3	22.8±4.7	17.8±2.9	10.7±1.7	8.4±0.9	6.4±0.5	6.5±0.8	6.4±0.7	5.8±0.5	5.9±0.6
	**80**	**120**	26.9±1.7	22.3±2.7	16.6±1.5	10.6±1.5	8.5±1.3	6.7±0.7	6.1±0.6	6.1±0.6	6.0±0.5	5.9±0.9
		**60**	26.7±1.9	22.7±2.1	16.6±1.9	10.8±1.3	8.5±1.2	6.6±0.5	6.0±0.7	6.3±0.7	5.8±0.5	5.8±0.5
		**30**	25.9±3.3	23.6±1.4	16.3±1.8	11.1±1.0	8.3±1.4	6.8±0.6	6.4±0.6	6.2±0.4	6.0±0.6	6.1±0.8
		**12**	26.9±1.7	22.3±2.7	16.6±1.5	10.6±1.5	8.5±1.3	6.7±0.7	6.1±0.6	6.1±0.6	6.0±0.5	5.9±0.9

FBP = filtered back-projection, IR = iterative reconstruction.

Data presented as mean ± standard deviation.

* p<0.05 vs. corresponding exposure settings with FBP.

^#^ p<0.05 vs. reference 120 kv 120 mAs with FBP or IR group (p-values for tests vs. other exposure parameters than reference not shown for sake of clarity).

**Table 5:** Total diameter for 11^th^ to 20^th^ airway generation (mm).

	[kV]	mAs	11th	12th	13th	14th	15th	16th	17th	18th	19th	20th
**FBP**	**120**	**120**	5.6±0.5	5.6±0.6	5.5±0.7	5.7±0.9	5.9±1.0	5.4±0.8	4.9±0.4	5.7±0.9	5.3±0.8	5.2±0.7
		**60**	5.4±0.5	5.9±1.1	5.6±0.9	5.6±0.7	5.9±1.0	5.3±0.5	5.3±0.7	5.2±1.3	5.4±0.9	5.6±0.8
		**30**	5.6±0.7	5.6±0.5	5.9±1.1	5.8±0.7	5.9±1.3	5.4±1.3	5.3±0.7	5.7±1.0	5.6±1.0	5.5±0.7
		**12**	6.1±1.5	5.9 ±0.7	5.7±0.8	5.7±0.9	6.0±1.1	5.7±1.0	5.9±1.0	5.4±1.2	5.2±1.3	5.4±0.2
	**80**	**120**	5.4±0.4	6.0±1.0	5.9±0.9	5.8±0.9	5.6±1.0	5.6±1.0	5.4±0.7	5.7±0.8	5.8±0.8	4.8±0.5
		**60**	5.6±0.4	6.3±2.2	5.6±0.6	5.9±1.0	5.8±1.0	5.7±1.0	5.4±1.1	5.7±1.0	5.2±0.7	5.3±0.4
		**30**	5.9±1.1	5.7±0.8	6.1±1.0	6.6±1.8	5.7±0.8	6.3±1.5	5.8±1.6	5.6±0.7	5.4±1.1	5.7±0.8
		**12**	6.0±1.2	6.2±0.9	6.2±1.3	6.0±1.0	6.5±1.0	6.7±1.8	5.3±1.4	5.6±1.2	5.8±0.8	5.4±0.6
**IR**	**120**	**120**	5.6±0.8	5.5±0.8	5.6±0.9	5.5±0.7	5.7±0.8	5.5±1.0	5.5±1.1	5.5±0.9	5.1±0.9	5.0±1.0
		**60**	5.4±0.5	5.7±0.8	5.6±1.0	6.1±1.2	5.5±0.9	5.2±0.3	5.5±0.9	5.6±0.8	5.4±0.5	5.3±0.6
		**30**	5.6±0.8	5.7±0.8	5.8±1.1	6.1±1.1	5.8±1.6	5.8±1.0	5.2±0.8	5.5±0.6	5.5±0.9	5.9±0.6
		**12**	5.6±0.5	5.7±0.8	5.7±0.7	5.5±1.1	6.0±1.6	5.8±1.2	5.9±1.4	5.7±1.2	4.9±0.9	5.6±1.0
	**80**	**120**	5.5±0.4	5.6±0.8	6.5±1.9	6.2±1.6	5.5±1.0	5.5±0.9	5.9±0.9	5.5±0.9	5.7±0.7	4.8±0.5
		**60**	5.5±0.7	5.4±1.2	5.6±0.6	5.8±0.9	5.7±1.3	5.5±0.9	5.1±0.8	4.7±0.8	6.0±0.8	5.4±1.0
		**30**	5.8±0.9	6.1±1.1	5.6±0.8	6.3±1.5	5.9±0.9	5.7±1.9	5.5±1.1	6.0±1.1	5.1±0.8	6.1±1.0
		**12**	5.5±0.4	5.6±0.8	6.5±1.9	6.2±1.6	5.5±1.0	5.5±0.9	5.9±0.9	5.5±0.9	5.7±0.7	4.8±0.5

FBP = filtered back-projection, IR = iterative reconstruction.

Data presented as mean ± standard deviation.

* p<0.05 vs. corresponding exposure settings with FBP.

^#^ p<0.05 vs. reference 120 kv 120 mAs with FBP or IR group (p-values for tests vs. other exposure parameters than reference not shown for sake of clarity).

In our study, TD from generations 9 to 15 are relatively constant and also from generations 16 to 20 (Tables 4 and 5). This holds true for WT also, which is a concession to the pig model with its monopodic branching pattern (Fig 1C).

### Influence of iterative reconstruction on airway parameters

There was no difference between FBP and IR for the most distal airway generation detected—with very few outliers–also neither within the number of detected airway segments, nor within the standard deviation (Table 1). Within the group TD 8 → 3 mm we made similar observations. Average WT% was stable between the two reconstruction algorithms with only one single outlier (Table 1). Within the group with TD 8 → 3 mm there were slightly but significantly higher values for WT% with FBP compared to IR in some reconstructions (Table 1). Pi10 was not affected by reconstruction algorithm. In the generation-based approach WT, WT%, and TD achieved similar values for FBP and IR at each reconstruction setting, with few unsystematic outliers (Tables 2–5).

## Discussion

Technical prerequisites need to be addressed when introducing quantitative post-processing of CT datasets to derive reproducible imaging biomarkers, which are potentially objective measures of disease severity. Reconstruction algorithms, slice thickness, radiation dose and also the type and manufacturer of the MDCT scanner influence image noise [11], as well as quantitative post-processing. Subsequently, the Sub-Populations and InteRmediate Outcome Measures In COPD Study (SPIROMICS) has proposed standardized CT acquisition protocols to allow reproducible quantitative post-processing among different centers [32]. In previous results from our center and also work by Hasegawa et al. [33], generation-based quantitative airway analysis was able to identify airway segments that correlate with the decline in lung function in cystic fibrosis [19], as well as in COPD [33]. In a further study by Hasegawa et al. [34] response to bronchodilatation therapy in COPD was also detected in airways corresponding to 4-6^th^ generation. Other indices normalizing wall thickness over several segmental and subsegmental airway generations have also been proven useful for quantifying airway disease [5, 35]. In this situation, segmentation and measurement of a large number of airways in the segmental and subsegmental quantitation by automatic quantitation tools is highly desirable. On the other hand, reduction of radiation dose for repeated examinations in clinical studies and therapy monitoring may potentially oppose exact automatic airway analysis by changing noise basically in two directions: 1.) influence on the detected airway segments and thus, inducing a sampling error, and 2.) a direct influence on the airway wall detection algorithm.

Previous studies have shown that reduced dose CT with IR has similar diagnostic quality to standard dose CT with FBP [36, 37]. For example Pontana et al. [13] showed that there was no difference in the objective noise, the contrast-to-noise ratio (CNR) and also signal-to-noise ratio (SNR) between standard-dose and low-dose CT with IR [13], showing that diagnostic CT at low-dose with IR may be similar to FBP and standard dose. Importantly, another more recent phantom study showed that the differentiation of low-contrast structures may be impaired using IR compared to FBP [38], which potentially also affects the differentiation of the airway wall in peripheral airways. Also, the SPIROMICS group specifically does not recommend IR in the setting of quantitative CT [32]. In previous studies using the *ex vivo* porcine chest phantom, we have addressed the influence of exposure settings and IR on detection and quantification of artificial pulmonary nodules, which is another typical application of chest CT post-processing [21, 22]. In the present study using the previously evaluated chest phantom, we sought to detect an influence of exposure settings and IR on quantitative CT of the airways.

Here, we observed only a slight but insignificant decrease in the absolute number of measured airway segments with reduced dose and an increase of standard deviation. Also, there was a tendency to a higher number of detected airway segments with IR compared to FBP at identical radiation dose (Table 1). These findings indicate, that noise indeed may influence the number of detected airway segments. On the other hand, the most distal airway generation detected remained stable across all exposure parameters. Next, we evaluated whether actual values for airway wall thickness, total diameter etc. were different among different exposure parameters.

In brief, our results indicate that fully automatic quantification of airway dimensions is not significantly influenced by exposure parameters as well as the IR technique across a vast range of airway generations, i.e. airway diameters (Tables 2–5). Further, the variations that we found in the study may be beyond clinical significance to date. These findings need to be put into perspective with few pre-existing studies on this objective. Mets et al. could show, that data obtained with IR were not inferior to FBP in measuring wall area (WA), lumen area (LA) and perimeter of airway in only one bronchus, the right upper lobe bronchus in 44 human smokers at low-dose settings [39]. Futher, Choo et al. [16] found a slight but significant difference in WT of the apical segmental bronchus within 281 patients but using only low dose chest CT comparing FBP and IR. Importantly, neither group analyzed different airway generations or different exposure parameters. Gomez-Cardona et al. [15] who used an airway phantom demonstrated that the standard deviation in measured WT was higher with decreasing radiation exposure and that IR could lower the difference between normal dose and lower dose. However, they did not compare the absolute WT between FBP and IR, but focused on relative bias and angular SD of WT. In our study we could not fully reproduce their observed difference in standard deviation for WT but we had similar results for standard deviation of airway segments detected. In contrast to Gomez-Cardona et al. [15] who used a synthetic phantom with 7 different artificial airways we made use of an *ex vivo* porcine chest phantom and analyzed all detectable airways per lung. Another reason for the different observations made in our study may result from a steady decline of TD through generation 8, but a fairly constant TD from Generation 9–15, whereas Gomez-Cardona et al. [15] found a continuous decrease in airway diameter as well as wall thickness measurements from trachea to subsegmental bronchi. This is due to the monopodial branching pattern in the pig lung as discussed below. We also used SAFIRE for IR reconstruction method as in previous studies [14, 21, 23] whereas they used MBIR.

Also, in contrast to the previous studies employing a limited number of airways in human subjects and limited variation in exposure settings, we were able to scan and analyze identical anatomical conditions repetitively at varying exposure settings from standard to low-dose, which is clearly not possible in patients [40, 41]. Further, we have shown previously that image noise and lung density in this *ex vivo* phantom are similar to the conditions in COPD and emphysema [21, 22].

We also have to point out some limitations of our study. It needs to be made clear that the software uses a priori anatomical knowledge and that it is trained to the human anatomy. The human bronchial tree has a bipodial dichotomic branching pattern which means that each bronchus divides in two subsequent bronchi, whereas in pigs and other mammals show a monopodial branching pattern, in which multiple bronchi arise from a longitudinal main bronchus. Monteiro et al. [42] showed that although pigs and humans have a different lung anatomy the tracheal diameter relationship did not show a big difference: the tracheal diameter in humans was only 1.2 times larger than in pigs, whereas in other mammals like rats the difference was 6.7 times larger. Our system YACTA is specifically adapted to human airway segmentation, and therefore the derived airway generations in pigs are not exactly identical to airway generations in a human lung. Because of different porcine anatomy we had to manually select a starting point in the trachea for automatic segmentation, otherwise there would have been segmentation problems also because of different anatomy. Thus, our software did not operate fully automatically, as in previous studies. However, as mentioned above, airway diameters and wall thicknesses encountered in the porcine airway phantom are comparable to those in the human situation. Our results are derived from one iterative reconstruction algorithm of one single vendor, and may not be readily transferred to other iterative reconstructions algorithms available. Also, after the completion of this study newer iterative reconstruction algorithms have become commercially available.

Consequently, this is the first report to compare generation based-airway analysis on FBP with IR images employing standard to low-dose MDCT. We measured an unprecedented number of airway segments including distal airway generations with a wall-thickness <1 mm, embedded in a realistic background. Our study demonstrates that there is no systematic difference in airway parameters between FBP and IR across standard-dose to low-dose acquisitions. Further, no systematic sampling error occurred because of missed airway segments. The results of the present study compare favorably to our previous results in this system, in which we could show that low-dose MDCT with IR is also not significantly influencing computer-aided lung nodule detection and volumetry [21, 22]. In conclusion, airway segmentation and quantification is robust over a wide range of exposure settings. Automatic airway analysis can be applied to low-dose MDCT with IR as it is recommended for clinical practice, in view of dose reduction in quantitative CT of airway disease.
